# An Insight into *Salvia haematodes* L. (Lamiaceae) Bioactive Extracts Obtained by Traditional and Green Extraction Procedures

**DOI:** 10.3390/plants11060781

**Published:** 2022-03-15

**Authors:** Rosa Tundis, Nicodemo Giuseppe Passalacqua, Marco Bonesi, Monica Rosa Loizzo

**Affiliations:** 1 Department of Pharmacy, Health and Nutritional Sciences, University of Calabria, 87036 Rende, Italy; marco.bonesi@unical.it (M.B.); monica_rosa.loizzo@unical.it (M.R.L.); 2 Museum of Natural History of Calabria and Botanic Garden, University of Calabria, 87036 Rende, Italy; nicodemo.passalacqua@unical.it

**Keywords:** *Salvia haematodes*, rapid solid–liquid dynamic extraction (RSLDE), extractor Naviglio^®^, Soxhlet apparatus, medium pressure liquid chromatography (MPLC), Alzheimer’s disease, antioxidant potential

## Abstract

Even though *Salvia* is one of the most known genera of the Lamiaceae family, some traditionally used *Salvia* species are still now less investigated. To that end, the present study aims to evaluate the chemical profile and the potential bioactivities of extracts and related fractions obtained from the endemic sage *Salvia haematodes* L. by applying a traditional extraction method such as Soxhlet apparatus (SHS) and the rapid solid–liquid dynamic extraction (RSLDE) by Naviglio extractor^®^ (SHN), considered among the “green techniques” operating at room temperature and with minimum solvent employment and minimum energy. Acetylcholinesterase (AChE) and butyrylcholinesterase (BChE) inhibitory activity was measured by a modified Ellman’s method. The antioxidant activity was investigated by using 2,2-diphenyl-1-picrylhydrazyl (DPPH), 2,2′-azino-bis (3-ethylbenzothiazoline-6-sulfonic acid) (ABTS), ferric reducing ability power (FRAP), and β-carotene bleaching tests. The SHN methanol fraction resulted the most active in all assays in particular in inhibiting lipid peroxidation with IC_50_ of 1.7 and 1.6 μg/mL, respectively, after 30 and 60 min of incubation. The SHN *n*-hexane fraction exhibited a selective activity against AChE with half-maximal inhibitory concentration (IC_50_) of 22.9 μg/mL, while the SHS *n*-hexane extract was more active against BChE (IC_50_ of 30.9 μg/mL). Based on these results, these fractions were subjected to further bio-fractionation by Medium Pressure Liquid Chromatography (MPLC) and the relative obtained fractions were investigated for their AChE and BChE inhibitory activity. A comparative analysis with bio-activity and chemical profile was performed. The observed biological effects provided us with a good starting point for further studies on *S. haematodes* extracts and fractions such as agents beneficial for the treatment of AD.

## 1. Introduction

*Salvia haematodes* L. (=*S. pratensis* L. subsp. *haematodes* (L.) Arcang.) belongs to *Salvia* subgen. *Sclarea* (Moench) Benth. (Lamiaceae) [1], and includes only herbaceous sages of the Old World having stamen with an elongated connective widening at the top. A phylogenetic analysis [2] revealed that this subgenus is included in a clade that is paraphyletic to subgen. *Salvia* L., showing a systematic distance between the typical sages (subgen. *Salvia*) and the sage under our investigation.

**Figure 1:**
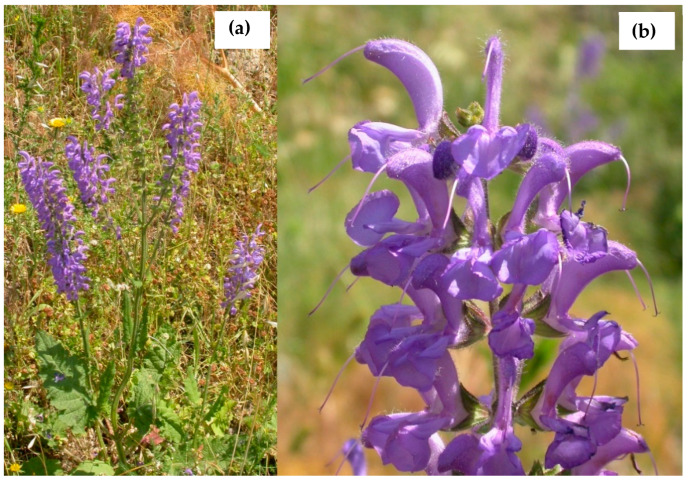
*Salvia haematodes* L. from Calabria (Southern Italy): (**a**) full plant in flower, and (**b**) inflorescence.

*S. haematodes* is a perennial herb with stems up to 100 cm, erect, branched, eglandular-pubescent below, glandular above. Basal leaves are simple, ovate, and long-petiolate (Figure 1). Cauline leaves are sessile and smaller. The inflorescences are from dense to lax, shortly branched with violet-blue flowers. *S. haematodes* is endemic to the Italian Peninsula, from the Marche to Calabria regions, where it grows as a hemicryptophyte scapose on Mediterranean hilly grasslands [3]. Few studies are present in the literature on this *Salvia* species. Its essential oil was predominantly characterized by the presence of monoterpenes, with sabinene as the most abundant constituent and significant amounts of borneol, 1,8-cineole, α-pinene, and (Z)-β-ocimene [4]. The main identified sesquiterpenes are γ-muurolene and β-caryophyllene. In another work, the ethanol extract of *S. haematodes* roots exhibited in vivo enhancement of wound healing, inhibition of carrageenin-induced pedal oedema, and induction of hypothermia [5]. Positive chronotropic and inotropic effects on isolated rabbit heart were also produced. *S. haematodes* ethanol extract demonstrated also in vivo analgesic properties, potentiation of pentobarbitone-induced narcosis, and antagonization of amphetamine-induced excitation [6].

*Salvia* species have been demonstrated to possess promising antioxidant, antimicrobial, anti-inflammatory, anti-mutagenic, and cytotoxic activities [7,8,9,10,11,12]. The protective effects in neurodegenerative diseases, including Alzheimer’s diseases (AD), of *Salvia* species have been also extensively assayed. Among them, there are *S. officinalis*, *S. leriifolia*, *S. glutinosa*, *S. argentea*, *S. indica*, *S. bracteata*, *S. quezelii*, *S. cryptantha*, *S. caespitosa*, *S. viscosa*, *S. microstegia*, *S. lavandulaefolia*, *S. multicaulis*, *S. fruticosa*, *S. pinnata*, *S. tobeyi*, and *S. syriaca*, [9,10,11,12,13]. *S. officinalis* and *S. lavandulaefolia* are the two *Salvia* species that showed beneficial effects by enhancing cognitive performance both in patients affected by dementia or cognitive impairment and healthy subjects. Additionally, both species were demonstrated to be safe with no serious adverse effects compared with placebo.

Alzheimer’s diseases (AD) is the most common cause of dementia in the ageing population, which leads to a progressive and irreversible decrease in mental function. The main degenerative condition is characterized by the formation of neurofibrillary tangles and amyloid plaques, and loss of neuron synapses.

Several studies shown early in the disease course a degeneration of cholinergic nuclei. Impairment of the cholinergic system is followed by alteration of attentional processes and cognitive decline. In fact, acetylcholine is a neurotransmitter found in many brain neurons that plays an important role in mental processes, such as memory and cognition. Moreover, it is implicated in smooth muscle contraction, dilatation of blood vessels, and increase in bodily secretions. Now, acetylcholinesterase (AChE) inhibitors represent the best treatment of AD [10]. In the brain, acetylcholine is hydrolysed by two cholinesterases, namely, acetylcholinesterase (AChE) and butyrylcholinesterase (BChE). It was found that in the brain of patients affected by AD, AChE is more abundant than BChE that contributes to the hydroxylation of acetylcholine in the cerebral cortex and hippocampus. However, it has been demonstrated that the activity of AChE is reduced by about 67% compared to the typical levels in the hippocampus and temporal lobe during the AD progression, whereas the activity of BChE was increased up to about 165% of the normal levels [13].

Initially, therapeutic approaches for improving cholinergic neurotransmission have focused on the use of AChE inhibitors, but successively several works demonstrated the relevance of both enzymes in the physio-pathology of AD, establishing the therapeutic importance to inhibit both AChE and BChE [13,14,15]. Cholinesterases inhibitors, preventing the degradation of acetylcholine, enhance the deficient brain cholinergic neurotransmission and are the first drugs authorized in Europe and the US for the symptomatic treatment of AD.

Numerous AChE inhibitors belong to the alkaloid class and include isoquinoline, steroidal, piperidine, indole, and quinolizidine alkaloids. Alkaloids are considered to be the most promising agents to treat AD due to their nitrogen containing structures. In fact, one of the AChE binding sites involves the interaction of the positively-charged nitrogen; although, another binding site has been found that allows the inhibition by non-alkaloid molecules. In fact, in recent years, different cholinesterases inhibitors with a non-alkaloidal structure have been isolated and tested. Among them, there are several flavonoids, phenolic compounds, coumarins, and several terpenes [16,17,18].

In this context and continuing our previous studies, the present work aimed to investigate, for the first time, the inhibitory capacity towards the enzymes playing a key role in neurodegenerative disorders, such as AD, of *S. haematodes* extracts in relation to their chemical profile. In this regard, the aerial parts of *S. haematodes* have been subjected to extraction by applying two different extraction procedures: Soxhlet apparatus and extractor Naviglio^®^. While the Soxhlet extraction is a classic exhaustive extraction technique widely applied to compounds that are sufficiently thermally stable, the rapid solid–liquid dynamic extraction (RSLDE) performed by extractor Naviglio^®^ is considered among the “green techniques” operating at room temperature and with minimum solvents employment and minimum energy. To the best of our knowledge, this is the first work in which this extraction procedure was applied to a *Salvia* species.

## 2. Results and Discussion

### 2.1. Biological Activity of S. haematodes Extracts

*S. haematodes* aerial parts, collected in southern Italy, were subjected to extraction by using two different extraction procedures with methanol such as a solvent: (1) Soxhlet apparatus (SHS) and (2) extractor Naviglio^®^ (SHN). Soxhlet extraction is a classic exhaustive extraction technique widely applied to compounds that are sufficiently thermally stable. The rapid solid–liquid dynamic extraction (RSLDE) performed by extractor Naviglio^®^ is considered among the “green techniques” operating at room temperature and with minimum solvents employment and minimum energy.

The extraction by using the extractor Naviglio^®^ is not carried out by osmosis or diffusion as occurs in most of the currently applied solid–liquid extraction techniques, but it is carried out by generating a negative pressure gradient between the internal and external sides of plant materials, followed by the restoration of the initial conditions of equilibrium. Solutions obtained by both extractive processes were combined and dried to obtain total extracts.

The extraction procedure that involves the use of the Soxhlet apparatus (SHS) has allowed obtaining a much higher extraction yield than that obtained with the extractor Naviglio^®^ (SHN). Values of 4.0 and 15.8% for SHN and SHS, respectively, were in fact obtained.

Successively, to operate a separation of non-polar compounds, both total extracts were re-suspended in methanol and partitioned with *n*-hexane. The *n*-hexane solutions were combined and dried to obtain *n*-hexane extracts (yield 2.3% of and 7.2% for SHN and SHS, respectively).

Total extracts, and methanol and *n*-hexane fractions of SHN and SHS were assessed for their potential AChE and BChE inhibitory properties. Inhibitors of cholinesterase were demonstrated to be one of the most promising and used agents to treat AD. The inhibition of both AChE and BChE enzymes has complimentary implications in the treatment of AD. In fact, it was shown that as AD progresses the AChE activity in certain brain regions declines, whereas the BChE activity increases in part to compensate for the loss in the AChE activity. *S. haematodes* extracts inhibited both enzymes in a concentration-dependent manner. IC_50_ (half maximal inhibitory concentration) values and SI (Selectivity Index) are reported in Table 1.

Both total extracts of SHN and all SHS showed a weak inhibitory activity against AChE with IC_50_ values of 396.4 and 249.8 μg/mL, respectively. Interestingly, SHS total extract showed a good inhibitory activity against BChE with an IC_50_ value of 35.8 μg/mL. However, if we analyse literature data, *S. haematodes* exhibited a more potent cholinesterase inhibitory activity than other *Salvia* species including, for example, *S. verbenaca* and *S. aegyptiaca* methanol and decoction that showed a percentage of inhibition lower than 40% at 100 μg/mL, respectively [19]. A similar observation should be made by comparing our data with data obtained by Topçu et al. [20] that investigated fourteen extracts of *Salvia* species against AChE and BChE. Among them, *S. chrysophylla* methanol extract resulted in the most active against AChE with the percentage of inhibition of 64.65% at 200 μg/mL followed by *S. staminea* (55.17%), whereas only *S. staminea* (methanol extract) and *S. poculata* (ethanol extract) exerted a percentage of BChE inhibitory activity greater than 50% at the maximum concentration tested.

After partitioning total extracts, the most active sample against AChE was the *n*-hexane fraction of SHN with an IC_50_ value of 22.9 μg/mL. Conversely, a promising activity against BChE was found for the *n*-hexane fraction of SHS with an IC_50_ value of 30.9 μg/mL. The *n*-hexane fraction of SHN could potently selectively inhibit AChE activities with a Selectivity Index (SI) of 17.8.

The different and selective activity towards the two enzymes can be explained by evaluating the different composition of the two fractions. For this reason, both SHN and SHS *n*-hexane fractions were analysed by gas chromatography–mass spectrometry (GC-MS). In Table 2 the main identified compounds are listed based on their Retention Index (RI) on HP-5 column.

A total of twenty-seven constituents were identified. SHN *n*-hexane fraction showed as dominant compounds neophytadiene (24.9%), β-sitosterol (20.1%), and stigmasterol (17.2%).

However, different fatty acid methyl esters and alkanes were identified. Moreover, SHS *n*-hexane fraction was characterized by neophytadiene as the most abundant compound (17.8%). However, this fraction was richer in fatty acid derivatives. Sterols were not identified. The sesquiterpene hydrocarbon *trans*-caryophyllene characterized both fractions with percentage of composition of 7.7% and 4.6% for SHN and SHS, respectively. Eugenol, γ-muurolene, and methyl myristate were not found in SHS fraction.

A close correlation between oxidative stress and degenerative diseases has been demonstrated [18,21,22]. In fact, it was demonstrated as oxidative stress is involved in the development and/or progression of AD by promoting tau hyper-phosphorylation, β-amyloids plaques deposition, and the consequent loss of synapses and neurons.

This relationship between oxidative stress and AD suggests the important part of oxidative stress in the pathological process; consequently, antioxidant agents may be useful for the treatment of AD [20]. Taking into account these considerations, we have decided to assess also the antioxidant effects of *S. haematodes* extracts. Data are reported in Table 3.

All samples exhibited antioxidant effects in a concentration-dependent manner. Generally, as might be expected for a higher concentration of polyphenolic compounds, the methanol fraction of both SHN and SHS, followed by the total extracts, evidenced the most promising activity. In particular, in the DPPH assay, all samples (IC_50_ values in the range 0.3–1.2 μg/mL), except the *n*-hexane fractions, were more active than the positive control ascorbic acid (IC_50_ value of 5.2 μg/mL).

The same trend was observed in the ABTS assay, but with IC_50_ values of 15.6 and 18.0 μg/mL for both SHN and SHS methanol fractions, respectively, and IC_50_ values of 28.1 and 42.1 μg/mL for the total extracts of SHN and SHS, respectively. However, all extracts are less active than the positive control ascorbic acid (IC_50_ value of 1.2 μg/mL). The ability of samples to induce a reduction in iron, assessed by applying FRAP test, revealed the following grade of potency in both SHN and SHS samples: MeOH fraction > Total extract > *n*-Hexane fraction. It is interesting to note that only SHN methanol fraction exhibited a ferric reducing ability power with a value of 70.3 μM Fe (II)/g greater than the positive control BHT (63.4 μM Fe (II)/g).

A promising ability to inhibit lipid peroxidation was evidenced by the β-carotene bleaching test for the methanol fraction of SHN with IC_50_ values of 1.7 and 1.6 μg/mL after 30 and 60 min of incubation, respectively. SHS samples were able to inhibit lipid peroxidation with IC_50_ values in the range 2.5–2.9 μg/mL. The greater antioxidant activity of SHN methanol fraction could be ascribed to the greater total phenol content (TPC) and total flavonoid content (TFC) that characterized this sample in comparison to the same extract obtained by Soxhlet extraction (Table 4).

In fact, methanol fraction obtained by using extractor Naviglio^®^ showed a TPC and a TFC of about two times and five times, respectively, higher than SHS.

In inhibiting lipid peroxidation, promising results were obtained with the *n*-hexane fraction also obtained by the Soxhlet apparatus with IC_50_ values of 2.8 and 2.9 μg/mL after 30 and 60 min of incubation, respectively. In this case, a weak activity was obtained with the *n*-hexane fraction of SHN with IC_50_ values of 7.7 and 14.0 μg/mL after 30 and 60 min of incubation, respectively.

### 2.2. Medium Pressure Liquid Chromatography (MPLC) Fractionation

Both *n*-hexane fractions obtained from SHN and SHS exhibited an interesting anti-cholinesterase activity. For this reason, these were subjected to fractionation. Briefly, a portion of the *n*-hexane fraction was subjected to medium pressure liquid chromatography (MPLC) on silica gel. This procedure afforded nine fractions from SHN (N1–N9) and seven fractions from SHS (S1–S7). All fractions were tested and the data are reported in Table 5. However, these fractions were shown to be less potent than *S. haematodes n*-hexane fractions, despite some interesting data.

The most active fractions against AChE were N9 and N1, with IC_50_ value of 39.5 and 40.2 μg/mL, respectively, while fractions N2-N8 showed weak activity. In the same group of fractions, against BChE interesting results were obtained with fractions N8 and N9 with IC_50_ values of 31.5 and 43.4 μg/mL. Among SHS samples, fraction S1 exhibited an interesting IC_50_ value of 40.4 μg/mL against AChE while the other fractions S2–S7 showed IC_50_ values in the range 90.9–458.7 μg/mL. Against BChE of interest are S2 and S3 fractions (IC_50_ value of 51.4 and 82.2 μg/mL, respectively).

N1, N8, N9, and S1, such as the most promising biologically active fractions, were analysed by GC-MS to identify the components potentially responsible for the enzymes inhibitory activity. Fractions were found to contain different phytochemical classes.

The main components of fraction N1 were sabinene, tetradecane, heptadecane, octadecane, neophytadiene, methyl myristate, and methyl stearate, while eugenol, *trans*-caryophyllene, α-humulene, docosane, tetracosane, pentacosane, and methyl palmitate were identified in fraction N8.

Fraction N9 was characterized by the presence of *trans*-caryophyllene, α-humulene, γ-muurolene, γ-cadinene, δ-cadinene, phytol, stigmasterol, β-sitosterol, heptacosane, octacosane, methyl linoleate, and methyl heptadecanoate as dominant constituents.

Methyl linoleate, methyl heptadecanoate, *trans*-caryophyllene, caryophyllene oxide, nonadecane, neophytadiene, and phytol were recognised in fraction S1.

Some compounds identified in *S. haematodes* bioactive fractions were assayed as pure molecules for their potential activity such as AChE and BChE inhibitors [23,24,25,26] (Table S1, Supplementary Materials). Among them, the diterpene alcohol phytol exhibited IC_50_ values of 12.51 and 23.89 μg/mL against AChE and BChE, respectively [25]. A weak activity was found for eugenol with IC_50_ values of 42.4 and 63.5 μg/mL against AChE and BChE, respectively [27]. Analyzing the inhibitory activity of AChE, molecular docking revealed that eugenol forms hydrogen bonds with Trp84 and Glu199 in the catalytic domain of this enzyme as well as a hydrogen bond with His 440 in the peripheral anionic site [28]. However, also the presence of aliphatic hydrocarbons and fatty acids, as well as phytosterols, resulted in AChE and BChE inhibition observed in our studied fractions.

Kayamoto et al. [29] showed the ability of the fatty acid methyl linoleate to exert a selective activity against AChE with an IC_50_ value of 68.8 μM, whereas a weak inhibitory activity was reported against BChE (IC_50_ of 247.8 μM). Not active was methyl palmitate against both enzymes.

Recently, phytosterols have gained the attention of researchers due to their anti-atherogenic properties, cholesterol and lipid lowering effects, and immune-modulating activity. A report on the changes in cholesterol metabolism of patients affected by AD was also present in literature [30].

Phytosterols are able to cross the blood–brain barrier and to accumulate in the brain. Therefore, they might play an important role in modulating pathways linked to neurodegeneration. Several studies have described the antioxidant and neuroprotective properties of β-sitosterol and stigmasterol [31,32,33,34,35]. β-Sitosterol has been demonstrated to increase the action of antioxidant enzymes by the stimulation of the estrogenic receptor/PI3-kinase-reliant pathway and to increase the levels of antioxidant enzymes in colon carcinogenesis. Lipid peroxidation and glucose oxidase-mediated oxidative stress have been inhibited through the incorporation of the phytosterol into the cell membrane [32]. β-sitosterol exhibited in vitro an anti-cholinesterase (with IC_50_ values of 55 and 50 μg/mL against AChE and BChE, respectively) and antioxidant activity and in vivo inhibited enzymes involved in the metabolism of cholinesterases and exerted radicals scavenging effects [30].

## 3. Materials and Methods

### 3.1. Chemicals and Reagents

Solvents of analytical grade were obtained from VWR International s.r.l. (Milan, Italy). Ascorbic acid, propyl gallate, butylated hydroxytoluene (BHT), 2,2-azinobis(3-ethylbenzothiazoline-6-sulfonic) acid (ABTS) solution, 2,2-diphenyl-1-picrylhydrazyl (DPPH), tripyridyltriazine (TPTZ), β-carotene, Tween 20, linoleic acid, acetylcholinesterase (AChE) from *Electrophorus electricus* (EC 3.1.1.7, Type VI-S) and butyrylcholinesterase (BChE) from equine serum (EC 3.1.1.8), acetylthiocholine iodide (ATCI), butyrylthiocholine iodide (BTCI), 5,5′-dithio-bis(2-nitrobenzoic acid) (DTNB), and physostigmine were purchased from Sigma-Aldrich S.p.a. (Milan, Italy).

### 3.2. Plant Materials

The aerial parts of S. haematodes were collected in Calabria (Southern Italy) in May 2012, along the county road SP 241, near the crossing to SP 197 (39.610744° N, 16.275371° E, WGS84, 60 m a.s.l.).

Plant materials were examined for integrity and absence of dust and insect contamination. Aerial parts were harvested in order to obtain an adequate quantity for the analysis. A voucher specimen (n. CLU 23976) was retained at the Natural History Museum of Calabria and the Botanic Garden, University of Calabria (Italy).

### 3.3. Extraction and Fractionation by Medium Pressure Liquid Chromatography (MPLC)

The air-dried and powdered aerial parts of *S. haematodes* were extracted with methanol by using:

(a) Soxhlet apparatus (SHS) (conventional glass with an extraction chamber with a diameter of 8 cm and a height of 30 cm, accompanied by a flask of capacity of 1 L; 600 mL, 8 extractive cycles);
(b) extractor Naviglio^®^ (SHN) (Nuova Estrazione S.a.s., Naples, Italy, 2 L capacity model; 30 extractive cycles each of which being 4 min). The combined extractive solutions were evaporated to dryness in vacuo using a rotary evaporator at 35–40 °C.

Yields of 2.3% and 15.8% were obtained for SHN and SHS, respectively. The total extracts were re-suspended in methanol (300 mL) and partitioned with *n*-hexane (7 × 260 mL). The combined solutions of *n*-hexane fraction were evaporated to dryness. A *n*-hexane fraction for SHN (yield of 4.0%) and a *n*-hexane fraction for SHS (yield of 7.2%) were obtained.

Both SHN and SHS *n*-hexane fractions, which exhibited an interesting anti-cholinesterase activity, were subjected to fractionation. Briefly, a portion of each extract was subjected to medium pressure liquid chromatography (MPLC) (Buchi Complete Flash System, column 920 mm x i.d. 26 mm, Specifications Pump Manager C-615, 2 Pump Modules C-605, Detector UV C-630, Fraction Collector Buchi C-660, Sepacore Record 1.0 Chromatography Software, adsorbent silica gel 20–45 mm, gradually increasing the eluent polarity from *n*-hexane/ethyl acetate 95:5 to ethyl acetate, flow rate 10 mL/min). This procedure afforded 9 fractions from SHN (N1–N9) and 7 fractions from SHS (S1–S7). All samples were stored at 4 °C for experimental use.

### 3.4. Chemical Analysis

The chemical composition of *S. haematodes* non-polar active fractions was assessed by using a Hewlett-Packard gas chromatograph (Agilent, Milan, Italy) equipped with a non-polar HP-5 capillary column (30 m × 0.25 mm, 0.25 μm), associated with a Hewlett-Packard mass spectrometer (Agilent, Milan, Italy) [36]. The ionization of the sample constituents was performed in electronic impact (EI, 70 electron volt). Helium was used as carrier gas (1.0 mL/min). The analyses were carried out as follows: isotherm at 50 °C for 5 min, temperature increase from 50 to 250 °C of 5 °C/min, and finally isotherm at 250 °C for 10 min. One microliter of diluted sample (1/10 *v*/*v*, in *n*-hexane) was injected. The identification of compounds was based on the comparison of their retention index (RI), either with those in literature or with those of available authentic standards (Sigma-Aldrich, Milan, Italy), on the comparison of the mass spectral data with the Wiley 138 library, and referring to the spectral data of pure compounds.

The methanol fractions were examined for their Total Phenol Content (TPC) and Total Flavonoid Content (TFC) as previously reported [36]. In brief, TPC was spectrophotometrically assessed by using Folin–Ciocalteu reagent. Absorbance was read at 765 nm using a UV–Vis Jenway 6003 spectrophotometer (Milan, Italy). In the TFC test, a method based on the formation of a flavonoid–aluminium complex was applied. Absorbance was read at 510 nm.

### 3.5. Analysis of Achetylcholinesterase (AChE) and Butyrylcholinesterase (BChE) Inhibitory Activity

The inhibition of AChE and BChE enzymes was measured by using a modified colorimetric Ellman’s method [36] based on the reaction of released thiocholine to give a coloured product with a chromogenic reagent such as AChE from *E. electricus* (EC 3.1.1.7, Type VI-S) and BChE equine serum (EC 3.1.1.8). Acetylthiocholine (ATCI) iodide and butyrylthiocholine iodide (BTCI) were employed as the substrates of the reaction. In brief, enzyme, essential oils, and phosphate buffer were mixed in microplates and incubated in an ice bath at 4 °C. After 30 min, physostigmine was added. The reaction started by adding 5,5′-dithiobis(2-nitrobenzoic-acid) (DTNB) solution and substrate. The microplate was placed in a thermostatic water bath (Branson model 3800-CPXH, Milan, Italy) for 20 min at 37 °C. The reaction was stopped by placing the microplate in an ice bath and adding physostigmine.

The absorbance was measured at 405 nm. Results are calculated as IC_50_ values (μg/mL).

### 3.6. In Vitro Antioxidant Tests

Four antioxidant assays were herein applied to investigate the antioxidant effects of *S. haematodes* extracts. The radicals scavenging activity was evaluated using two spectrophotometric methods, such as DPPH and ABTS tests according to the procedure previously reported [37]. DPPH is a radical characterized by an intense purple colour, known for its stability due to the delocalization of the radical in aromatic rings. In this assay, the radical is neutralized by accepting either a hydrogen atom or an electron from an antioxidant agent or a reducing agent. When an odd electron pairs up with another electron, the initial colour gradually decolorizes into pale yellow. In the test, in brief, the DPPH solution (1.0 × 10^−4^ M) and *S. haematodes* extracts at different concentrations (in the range from 62.5 to 1000 μg/mL) were mixed. After 30 min, the absorbance was read at 517 nm. Compared to DPPH, which is stable by nature, the ABTS^+^ radical is a radical that should be generated by chemical reactions. When this radical (unstable form) accepts an electron from an antioxidant agent, the blue-green colour changes into a pale blue colour, which is the regeneration of the stable form of ABTS. To obtain ABTS radical cation solution (ABTS^+^), ABTS solution (7 mM) and potassium persulphate (2.45 mM) were mixed. After 12 h, ABTS^+^ was diluted with ethanol to final absorbance of 0.70 at 734 nm. Successively, 2 mL of diluted ABTS^+^ solution was added to extracts (25 μL) at concentrations in the range 1–400 μg/mL. After 6 min, the absorbance was read at 734 nm. In both DPPH and ABTS tests, ascorbic acid was used as a positive control.

The ability to reduce iron ions was assessed using FRAP test [37]. A solution of tripyridyltriazine (TPTZ), FeCl_3_, HCl, and acetate buffer at pH 3.6 was prepared in order to obtain the FRAP reagent.

The FRAP reagent (2.0 mL), water (900 μL), and *S. haematodes* samples (100 μL at the concentration of 2.5 mg/mL) were mixed. After 30 min of incubation, the absorbance was measured at 595 nm. Butylated hydroxytoluene (BHT) was used as a positive control.

The capacity of *S. haematodes* aerial parts to protect lipid peroxidation was analysed by applying the β-carotene bleaching test, as previously reported [37]. Briefly, a mixture of β-carotene, linoleic acid, and 100% Tween 20 was prepared and the obtained emulsion was added to a 96-well microplate containing samples at concentrations in the range 2.5–100 μg/mL. The absorbance was measured at 470 nm against a blank at t = 0 and after 30 and 60 min of incubation. Propyl gallate was used as a positive control.

### 3.7. Statistical Analysis

The concentration giving 50% inhibition (IC_50_) was obtained by nonlinear regression by using Prism GraphPad Prism version 4.0 for Windows (San Diego, CA, USA). The concentration–response curve was obtained by plotting the percentage inhibition vs. concentration. Differences within and between groups were evaluated by One-way analysis of variance test (ANOVA) followed by a multi-comparison Dunnett’s test, used to compare each group with the positive control.

## 4. Conclusions

*Salvia* species have been traditionally used for the treatment of several ailments, with particular reference to cognitive and neurological conditions [38].

The analysis of literature confirms that many *Salvia* species and their main bioactive constituents influence some biological processes that may have an important impact on cognitive and neurological functions. Currently, *S. officinalis* and *S. lavandulaefolia* are the two *Salvia* species investigated in human studies, so the potency and the efficacy of other *Salvia* species are uncertain and deserve to be better investigated. Among these is *S. haematodes*.

To the best of our knowledge, this work is the first report on the AChE and BChE inhibitory activity of this *Salvia* species. In our study, the aerial parts of this *Salvia* species were extracted by using two different extraction procedures with methanol as a solvent, such as Soxhlet apparatus and extractor Naviglio^®^ in order to investigate the impact on the extract composition and, consequently, on their biological effects. Despite the use of the traditional extraction technique of the Soxhlet apparatus, we obtained a better extraction yield, by analysing the antioxidant activity of samples it is evident that the methanol fraction obtained by extractor Naviglio^®^ has a better antioxidant activity in all the carried-out tests, data that are supported by the higher content of TPC and TFC. This is not valid when we examine data relating to the cholinesterase inhibitory activity of the *n*-hexane extracts. In this case, in fact, the SHN *n*-hexane extract was more active against AChE, while the SHS *n*-hexane extract was more active on BChE. The bio-fractionation of these *n*-hexane fractions leads to less active samples. This anti-cholinesterase activity may be due to synergistic effects shown by the components of the mixtures in the test system used in this investigation.

Results obtained in this work provide the basis for further studies, necessary to confirm the activity of *S. haematodes* extracts and related fractions and the identification of some biologically active compounds. Several species of *Salvia* are commonly ingested across numerous cultures, which increases confidence about its safety. Moreover, in this case, further confirmation about the safety of *S. haematodes* is necessary.

## Figures and Tables

**Table 1:** Cholinesterases (AChE and BChE) inhibitory activity (IC_50_ μg/mL) of *S. haematodes*.

*S. haematodes*	Extract/Fraction	AChE	BChE	SI ^#^
SHN	Total	396.4 ± 3.4 ^a^	559.9 ± 5.2 ^a^	1.4
	Methanol	4.5% ^#^	33.1% ^#^	-
	*n*-Hexane	22.9 ± 1.0 ^a^	408.4 ± 3.8 ^a^	17.8
SHS	Total	249.8 ± 2.7 ^a^	35.8 ± 0.9 ^a^	0.1
	Methanol	458.2 ± 4.2 ^a^	312.1 ± 3.4 ^a^	0.7
	*n*-Hexane	214.2 ± 3.2 ^a^	30.9 ± 1.1 ^a^	0.1
Physostigmine		0.2 ± 0.02	2.4 ± 0.04	12

Data are expressed as mean ± S.D. (*n* = 3). SHN: *S. haematodes* aerial parts extracted by extractor Naviglio^®^; SHS: *S. haematodes* aerial parts extracted by Soxhlet apparatus. ^#^ SI (Selectivity Index): IC_50_ BChE/IC_50_ AChE. ^#^ percentage of inhibition at the concentration of 500 µg/mL. AChE and BChE tests: One-way ANOVA *** *p* < 0.0001 followed by a multicomparison Dunnett’s test: ^a^ *p* < 0.01 compared with physostigmine.

**Table 2:** The main constituents (%) of *S. haematodes n*-hexane fractions.

Compound	Class	RI ^a^	SHN	SHS	I.M. ^b^
Sabinene	MH	973	1.3 ± 0.04	tr	1, 2, 3
Eugenol	PH	1354	0.7 ± 0.02	-	1, 2
*trans*-Caryophyllene	SH	1415	7.7 ± 2.3	4.6 ± 0.7	1, 2, 3
α-Humulene	SH	1455	1.5 ± 0.2	tr	1, 2
γ-Muurolene	SH	1478	0.3 ± 0.01	-	1, 2
γ-Cadinene	SH	1515	0.4 ± 0.05	0.3 ± 0.04	1, 2
δ-Cadinene	SH	1526	1.4 ± 0.01	0.5 ± 0.06	1, 2
Caryophyllene oxide	OS	1580	tr	3.5 ± 1.0	1, 2
Neophytadiene	DI	1837	24.9 ± 2.3	17.8 ± 1.5	1, 2
Phytol	DI	2111	2.5 ± 1.2	1.4 ± 0.7	1, 2
Methyl myristate	FA	1726	0.9 ± 0.02	-	1, 2
Methyl palmitate	FA	1928	1.7 ± 0.3	6.7 ± 0.7	1, 2
Methyl linoleate	FA	1996	3.1 ± 0.4	2.4 ± 0.03	1, 2
Methyl heptadecanoate	FA	2030	1.2 ± 0.2	1.8 ± 0.1	1, 2
Methyl stearate	FA	2128	1.8 ± 0.1	3.7 ± 0.1	1, 2
Tetradecane	AL	1400	0.7 ± 0.02	2.9 ± 0.1	1, 2, 3
Heptadecane	AL	1700	0.5 ± 0.03	tr	1, 2, 3
Octadecane	AL	1800	0.7 ± 0.01	0.4 ± 0.05	1, 2, 3
Nonadecane	AL	1900	0.3 ± 0.01	3.5 ± 0.5	1, 2, 3
Docosane	AL	2200	1.1 ± 0.02	1.0 ± 0.06	1, 2, 3
Tetracosane	AL	2400	1.2 ± 0.01	tr	1, 2, 3
Pentacosane	AL	2500	1.4 ± 0.2	8.6 ± 0.7	1, 2, 3
Heptacosane	AL	2700	1.8 ± 0.04	1.0 ± 0.1	1, 2, 3
Octacosane	AL	2800	0.9 ± 0.01	1.3 ± 0.01	1, 2, 3
Stigmasterol	ST		17.2 ± 1.5	5.4 ± 0.9	1, 2
β-Sitosterol	ST		20.1 ± 2.1	2.3 ± 0.1	1, 2

Data are reported as the mean ± standard deviation (*n* = 3). tr: traces. -: not identified. ^a^ RI: Retention indices on the HP-5 column. ^b^ IM, identification method: 1: Cmparison of retention times; 2: Comparison of mass spectra with MS libraries, 3: Comparison with authentic compounds. Monoterpene Hydrocarbons: MH; Sesquiterpene Hydrocarbons: SH; Oxygenated Sesquiterpenes: OS; Phenolic compound: PH; Diterpenes: DI; Fatty acids derivatives: FA; Sterols: ST.

**Table 3:** *In vitro* antioxidant activity of *S. haematodes*.

*S. haematodes*		DPPH Test (IC_50_ μg/mL)	ABTS Test (IC_50_ μg/mL)	FRAP Test * (μM Fe(II)/g)	β-Carotene Bleaching Test (IC_50_ μg/mL)	
	Extract/Fraction				30 min	60 min
SHN	Total	0.4 ± 0.07 ^a^	28.1 ± 1.1 ^a^	49.6 ± 1.7 ^a^	5.3 ± 0.05 ^a^	7.9 ± 0.08 ^a^
	*n*-Hexane	17.9 ± 0.8 ^a^	81.1 ± 2.8 ^a^	10.3 ± 0.9 ^a^	7.7 ± 0.02	14.0 ± 0.02
	Methanol	0.3 ± 0.03 ^a^	15.6 ± 1.3 ^a^	70.3 ± 2.8 ^a^	1.7 ± 0.09 ^a^	1.6 ± 1.5 ^a^
SHS	Total	1.2 ± 0.02 ^b^	42.1 ± 1.9 ^a^	48.7 ± 1.7 ^a^	2.9 ± 0.05 ^a^	2.6 ± 0.05
	*n*-Hexane	41.9 ± 1.6 ^a^	220.5 ± 2.5 ^a^	1.2 ± 0.05 ^a^	2.8 ± 0.03 ^a^	2.9 ± 0.07 ^c^
	Methanol	0.9 ± 0.04 ^b^	18.0 ± 0.9 ^a^	58.7 ± 1.2 ^b^	2.7 ± 0.02 ^a^	2.5 ± 0.04
Ascorbic acid		5.2 ± 0.8	1.2 ± 0.03	-		
BHT		-	-	63.4 ± 4.5		
Propyl gallate					1.3 ± 0.04	1.2 ± 0.03

Data are expressed as mean ± S.D. (*n* = 3). * Samples tested at the concentration of 2.5 mg/mL. DPPH test: One-way ANOVA *** *p* < 0.0001 followed by multicomparison Dunnett’s test: ^a^ *p* < 0.01 compared with ascorbic acid, ^b^ *p* < 0.05 compared with ascorbic acid; ABTS test: One-way ANOVA *** *p* < 0.0001 followed by multicomparison Dunnett’s test: ^a^ *p* < 0.01 compared with ascorbic acid; FRAP test: One-way ANOVA *** *p* < 0.0001 followed by multicomparison Dunnett’s test: ^a^ *p* < 0.01 compared with ascorbic acid, ^b^ *p* < 0.05 compared with BHT; β-Carotene bleaching test (t = 30 min): One-way ANOVA *** *p* < 0.0001 followed by multicomparison Dunnett’s test: ^a^ *p* < 0.01 compared with propyl gallate; β-Carotene bleaching test (t = 60 min): One-way ANOVA *** *p* < 0.0001 followed by multicomparison Dunnett’s test: ^a^ *p* < 0.01 compared with ascorbic acid, ^c^ *p* > 0.05 compared with propyl gallate.

**Table 4:** Total phenol content (TPC) and total flavonoid content (TFC) of *S. haematodes*.

*S. haematodes* Methanol Fraction	TPC ^a^	TFC ^b^	TFC/TPC
SHN	55.2 ± 1.3	36.5 ± 1.0	0.7
SHS	29.0 ± 1.1	7.2 ± 0.5	0.2

Data are expressed as mean ± S.D. (*n* = 3). ^a^ mg of chlorogenic acid equivalents (CA)/g of plant materials. ^b^ mg of quercetin equivalents (QE)/gram of plant materials.

**Table 5:** AChE and BChE inhibitory activity (IC_50_, μg/mL) of *S. haematodes* SHN and SHS fractions.

*S. haematodes*	Fraction	AChE	BChE	SI
SHN	N1	40.2 ± 1.5 ^a^	61.5 ± 1.6 ^a^	1.5
	N2	180.7 ± 4.2 ^a^	166.3 ± 2.4 ^a^	0.9
	N3	62.1 ± 2.6 ^a^	73.9 ± 2.2 ^a^	1.2
	N4	564.9 ± 3.4 ^a^	129.8 ± 2.5 ^a^	0.2
	N5	115.2 ± 1.8 ^a^	52.7 ± 1.3 ^a^	0.5
	N6	408.6 ± 3.5 ^a^	53.9 ± 1.6 ^a^	0.1
	N7	141.9 ± 2.8 ^a^	55.9 ± 1.5 ^a^	0.3
	N8	110.0 ± 2.0 ^a^	31.5 ± 1.0 ^a^	0.3
	N9	39.5 ± 1.2 ^a^	43.4 ± 1.1 ^a^	1.1
SHS	S1	40.4 ± 1.1 ^a^	112.6 ± 2.4 ^a^	2.8
	S2	114.4 ± 3.1 ^a^	51.4 ± 1.1 ^a^	0.4
	S3	90.9 ± 2.2 ^a^	82.2 ± 1.4 ^a^	0.9
	S4	418.7 ± 3.6 ^a^	207.7 ± 4.4 ^a^	0.5
	S5	444.8 ± 3.9 ^a^	167.0 ± 3.5 ^a^	0.4
	S6	458.7 ± 3.7 ^a^	110.1 ± 1.2 ^a^	0.2
	S7	307.8 ± 2.8 ^a^	173.8 ± 4.0 ^a^	0.6
Physostigmine		0.2 ± 0.02	2.4 ± 0.04	12

Data are expressed as mean ± S.D. (*n* = 3). SI: IC_50_ BChE/IC_50_ AChE. AChE and BChE tests: One-way ANOVA *** *p* < 0.0001 followed by multicomparison Dunnett’s test: ^a^ *p* < 0.01 compared with physostigmine.

## Supplementary Materials

The following supporting information can be downloaded at: https://www.mdpi.com/article/10.3390/plants11060781/s1, Table S1. AChE and BChE inhibitory activity (IC50, μg/mL and/or μM) of pure compounds from literature.
