# Supplementary material for: An Insight into Salvia haematodes L. (Lamiaceae) Bioactive Extracts Obtained by Traditional and Green Extraction Procedures
Source: Plants (Basel). 2022 Mar 15;11(6):781. doi: 10.3390/plants11060781 (PMC8956035; doi:10.3390/plants11060781)
Supplement: Supplementary file 1 [file plants-11-00781-s001.zip › 1.pdf]

Supplementary Materials

Table S1. AChE and BChE inhibitory activity (IC_{50}, µg/mL and/or µM) of pure compounds from literature.

Compound	Fraction	AChE	BChE	Ref.
trans-Caryophyllene	N8, N9, S1	32% (at 0.06 mM)	78.6	21, 22
Phytol	N9, S1	12.5 µg/mL	23.9 µg/mL	23
Caryophyllene oxide	S1	41.46 (at 200 µg/mL)	61.03 (at 200 µg/mL)	24
Eugenol	N8	42.4 µg/mL	63.5 µg/mL	25
Methyl linoleate	N9, S1	68.8 µM	247.8 µM	27
Methyl palmitate	N8	> 500 µM	> 500 µM	27
β-Sitosterol	N9	55.0 µg/mL	50.0 µg/mL	29
Stigmasterol	N9	63.0 µg/mL	67.0 µg/mL	33
